# Evaluation of the performance of deformable image registration between planning CT and CBCT images for the pelvic region: comparison between hybrid and intensity-based DIR

**DOI:** 10.1093/jrr/rrw123

**Published:** 2017-02-02

**Authors:** Yoshiki Takayama, Noriyuki Kadoya, Takaya Yamamoto, Kengo Ito, Mizuki Chiba, Kousei Fujiwara, Yuya Miyasaka, Suguru Dobashi, Kiyokazu Sato, Ken Takeda, Keiichi Jingu

**Affiliations:** 1 Department of Radiation Oncology, Tohoku University Graduate School of Medicine, 1-1 Seiryo-machi, Aoba-ku, Sendai 980-8574, Japan; 2 Department of Radiological Technology, Graduate School of Health Sciences, Faculty of Medicine, Tohoku University, 1-1 Seiryomachi, Aoba-ku, Sendai 980-8574, Japan; 3 Radiation Technology, Tohoku University Hospital, 1-1 Seiryo-machi, Aoba-ku, Sendai 980-8574, Japan

**Keywords:** radiotherapy, deformable image registration, dose accumulation, CBCT, prostate cancer

## Abstract

This study aimed to evaluate the performance of the hybrid deformable image registration (DIR) method in comparison with intensity-based DIR for pelvic cone-beam computed tomography (CBCT) images, using intensity and anatomical information. Ten prostate cancer patients treated with intensity-modulated radiation therapy (IMRT) were studied. Nine or ten CBCT scans were performed for each patient. First, rigid registration was performed between the planning CT and all CBCT images using gold fiducial markers, and then DIR was performed. The Dice similarity coefficient (DSC) and center of mass (COM) displacement were used to evaluate the quantitative DIR accuracy. The average DSCs for intensity-based DIR for the prostate, rectum, bladder, and seminal vesicles were 0.84 ± 0.05, 0.75 ± 0.05, 0.69 ± 0.07 and 0.65 ± 0.11, respectively, whereas those values for hybrid DIR were 0.98 ± 0.00, 0.97 ± 0.01, 0.98 ± 0.00 and 0.94 ± 0.03, respectively (*P* < 0.05). The average COM displacements for intensity-based DIR for the prostate, rectum, bladder, and seminal vesicles were 2.0 ± 1.5, 3.7 ± 1.4, 7.8 ± 2.2 and 3.6 ± 1.2 mm, whereas those values for hybrid DIR were 0.1 ± 0.0, 0.3 ± 0.2, 0.2 ± 0.1 and 0.6 ± 0.6 mm, respectively (*P* < 0.05). These results showed that the DSC for hybrid DIR had a higher DSC value and smaller COM displacement for all structures and all patients, compared with intensity-based DIR. Thus, the accumulative dose based on hybrid DIR might be trusted as a high-precision dose estimation method that takes into account organ movement during treatment radiotherapy.

## INTRODUCTION

Development of the intensity-modulated radiotherapy (IMRT) technique has enabled the tailoring of dose distributions to complex target shapes and allowed us to increase the dose to the planning target volume (PTV) and decrease the dose to organs at risk [1–6]. However, the intricate dose distribution is more susceptible to position and anatomy uncertainties of the tumor and normal tissues during the course of treatment radiotherapy [7–10]. Previous studies have shown that pelvic anatomy such as the prostate, rectum, bladder, or seminal vesicles change during the course of radiotherapy [11–15]. These interfraction variations in prostate IMRT cause dosimetric effects [16].

In order to estimate the accumulative dose over the treatment days, deformable image registration (DIR) has been used. Previous studies have reported that to evaluate the dose delivered to the targets and organs at risk (OARs) in the set-up and anatomical change, DIR is required in order to calculate the accumulative dose on the reference image [using repeat computed tomography (CT) or cone-beam CT (CBCT) images] [17–20]. If a CBCT image is acquired for patient set-up for each fraction, we can calculate the accumulative dose using repeated CBCT images with no additional exposure for the patient.

Commercial DIR software [including MIM Maestro (MIM Software Inc., Cleveland, USA), Velocity (Varian Medical Systems, Palo Alto, USA) and RayStation (RaySearch Laboratories, Stockholm, Sweden] is available for use in clinical practice to calculate the accumulative dose using CBCT images [21–23]. In these software packages, an intensity-based DIR algorithm (i.e. DIR without structural information) is commonly used. It is expected that this DIR would not result in good registration accuracy when the deformation is large or when the boundaries between structures are not clear [24, 25]. It is expected that the pelvic region would have larger interfractional organ motion than other clinical sites (e.g. the head and neck region) [26]. To improve DIR accuracy, hybrid DIR has been recently developed and implemented in RayStation [27]. This has the potential to improve DIR accuracy, even with large interfractional organ motion [28]. Till now there has been no data showing the performance of hybrid DIR between planning CT and CBCT for the pelvic region.

In this study, we evaluated the performance of commercially available hybrid DIR between planning CT and CBCT in comparison with intensity-based DIR for the pelvic region.

## MATERIAL AND METHODS

### Patient data and image materials

This study received approval from our institutional reviewer board (2015-1-167). Ten prostate cancer patients treated with IMRT in our hospital were studied. No additional selection criteria were used. All patients had gold fiducial markers implanted in the prostate for daily set-up correction according to our clinical IMRT protocol. In addition, prior to the acquisition of the treatment planning CT images and 30 min before the daily IMRT, each patient urinated to ensure the bladder was in the same state. In addition, the patients emptied their bowels just before the daily IMRT. Details are described elsewhere [29].

Planning CT images of each patient were acquired within the 14 days before treatment. All planning CT images were obtained with a Light Speed RT16 unit (GE Medical Systems, Waukesha, WI). The kV on-board imager (OBI) version 1.5 system integrated in a model 23EX linear accelerator (Varian Medical Systems, PaloAlto, CA) was used to acquire CBCT images. Full-fan mode with a half bow-tie filter was used for acquisition of all CBCT images (pelvic mode) [30]. Each CBCT image was acquired on the first treatment day and then approximately every 4 days (a total of 9–10 scans per patient). For this study, an experienced radiation oncologist contoured the prostate, the rectum, the bladder, and the seminal vesicles on the planning CT and on each CBCT scan.

### Deformable image registration

Before performing DIR, all the CBCT images were rigidly registered in the corresponding planning CT image by matching the gold fiducial makers, using Eclipse. In clinical practice, we did manual rigid registration between the CBCT images and the planning CT image by matching the gold fiducial markers, using Eclipse. Then, we exported all the CBCT images, which had already been registered, to RayStation. This is because DIR can only be performed on image sets that already have a rigid registration in RayStation version 4.5.1 (RaySearch Laboratories, Stockholm, Sweden). Then, all CBCT images were deformed to the corresponding planning CT images, using hybrid intensity and structure-based DIR (the ANACONDA algorithm) implemented in RayStation. This algorithm combines image information (i.e. intensities) with anatomical information as provided by contoured image sets [27]. This algorithm is based on a mathematical formula in which the registration is a non-linear optimization problem. The object function was (i) to maintain image similarity, (ii) to keep the image grid smooth and invertible, (iii) to keep the deformation anatomically reasonable when structures are present and (iv) a penalty term when structures are used [23]. In the process of DIR optimization, the weight ratio between image intensity and contour information is same weight (i.e. 0.5 vs 0.5), which is default in RayStation. To evaluate the effect of an approach that combines the intensity-based and anatomical information–based approaches for the improvement of DIR accuracy, two different DIR parameter settings were employed. One setting used only the whole body structure as controlling region of interest (ROI) for DIR. This setting assumes that this registration uses the intensity-based approach (i.e. intensity-based DIR). The other setting used the prostate, rectum, bladder, seminal vesicles, and body structures as controlling ROIs for DIR, assuming that this registration is based on both the intensity-based and anatomical information-based approaches (i.e. hybrid DIR). DIR was performed between the planning CT and each CBCT to obtain the deformation vector field (DVF). Then, the DVFs were applied to contours of the prostate, rectum, bladder, and seminal vesicles on each CBCT to validate the level of DIR accuracy.

### Evaluation of DIR accuracy

A Dice similarity coefficient (DSC), which measures the overlap between each pair of contours, was used to quantitatively evaluate the DIR accuracy [31]. The DSC ranges from zero to one (indicating no overlap and a perfect agreement, respectively) and is calculated using:

$$\text{Dice}\;\text{similarity}\;\text{coeffcient}=\frac{V_{\mathrm{reference}}\cap V_{\mathrm{deformed}}}{\frac{V_{\mathrm{reference}}+V_{\mathrm{deformed}}}{2}},$$

where V_reference_ and V_deformed_ are the volumes of the contour on the planning CT and the contour deformed from the CBCT on the planning CT, respectively.

In addition, center of mass (COM) displacement between the planning contour and the deformed contour was used to validate the DIR accuracy for the deformed organ contour. The evaluated structures were the prostate, the rectum, the bladder, and the seminal vesicles. A paired *t*-test was used with JMP, version 11.2.0, software (SAS Institute, Cary, NC) to test whether the Dice similarity coefficient and COM distance of the hybrid DIRs were significantly higher than that of intensity-based DIRs (*P* < 0.05).

## RESULTS

**Fig. 1.**
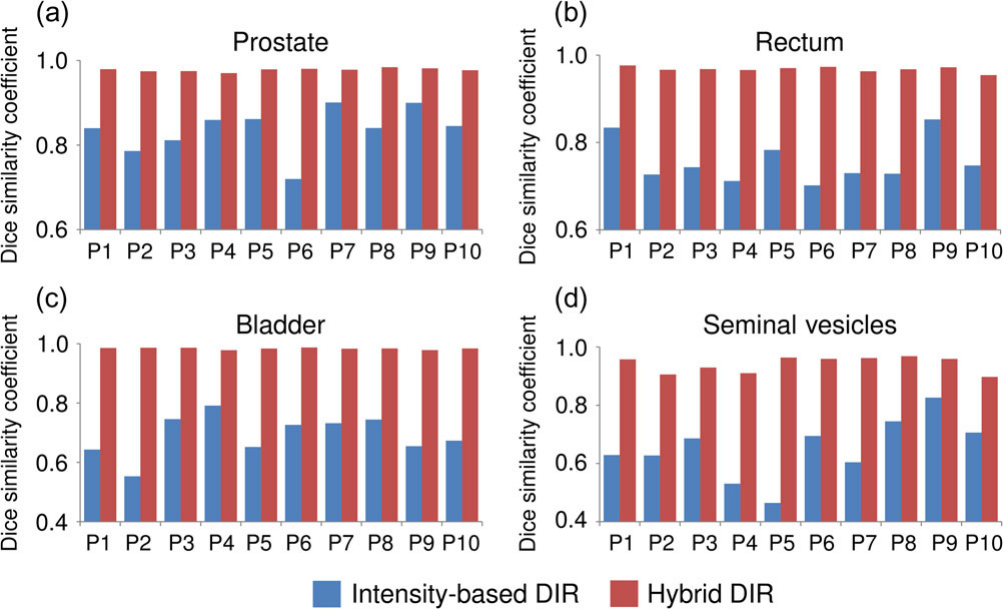
Dice similarity coefficient between the manual contour on the planning CT and the deformed contour by two different DIR methods for each patient in the prostate, rectum, bladder, and seminal vesicles, respectively.

**Fig. 2.**
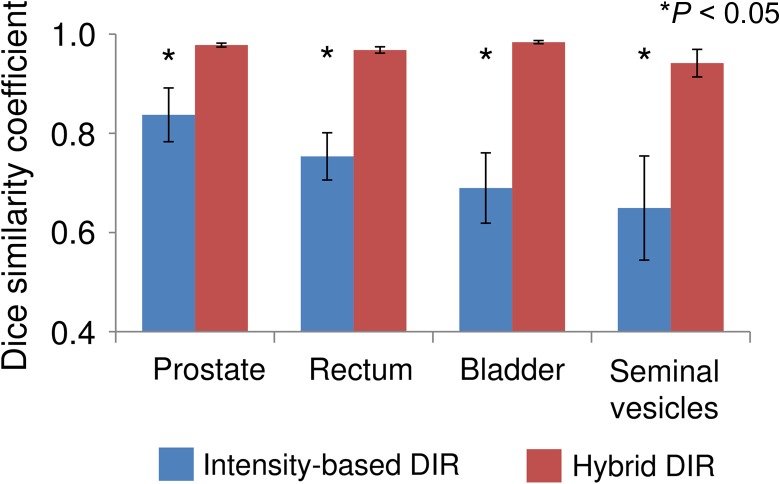
Average dice similarity coefficient between the manual contour on the planning CT and the deformed contour by two different DIR methods for all ten patients for prostate, rectum, bladder, and seminal vesicles.

**Fig. 3.**
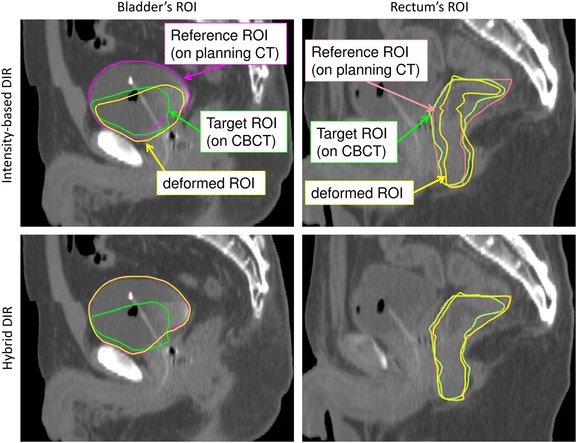
Typical example of rectum and bladder structures based on each DIR (Patient 2). Purple lines: the manual bladder structure on planning CT; green lines: the manual structure on CBCT; yellow lines: the structure deformed by DIR; orange lines: the manual rectum structure on planning CT.

Figure 1 shows the DSCs in intensity-based DIR and hybrid DIR for the prostate, rectum, bladder, and seminal vesicles in 10 patients. For intensity-based DIR, the DSCs varied widely with patients for all structures. On the other hand, for hybrid DIR, there were small variations in DSC among patients for all structures. Figure 2 shows the average DSCs over all cases. The average DSCs for intensity-based DIR for the prostate, rectum, bladder, and seminal vesicles were 0.84 ± 0.05, 0.75 ± 0.05, 0.69 ± 0.07 and 0.65 ± 0.11, respectively, whereas those values for hybrid DIR were 0.98 ± 0.00, 0.97 ± 0.01, 0.98 ± 0.00 and 0.94 ± 0.03, respectively. These results showed that hybrid DIR had a higher DSC value than intensity-based DIR for all structures. Table 1 shows the summary of the COM distance for each structure. Average COM displacements for intensity-based DIR for the prostate, rectum, bladder, and seminal vesicles were 3.1 ± 1.5, 4.1 ± 1.4, 7.9 ± 2.2 and 3.6 ± 1.2 mm, whereas those values for hybrid DIR were 0.1 ± 0.0, 0.3 ± 0.2, 0.2 ± 0.1 and 0.6 ± 0.6 mm, respectively (*P* < 0.05). These results showed that hybrid DIR had smaller COM displacements than intensity-based DIR for all structures. Figure 3 shows an example case of deformed structures using two DIR algorithms (Patient 2). By visual inspection, hybrid DIR could deform the rectum and bladder accurately, compared with intensity-based DIR [DSC of rectum: 0.65 (intensity-based DIR) vs 0.96 (hybrid DIR); DSC of bladder: 0.49 (intensity-based DIR) vs 0.99 (hybrid DIR)].

**Table 1.** Summary of center of mass displacement with a standard deviation (mm) for prostate, rectum, bladder, and seminal vesicles

Patient number	Prostate		Rectum		Bladder		Seminal vesicles	
	Intensity	Hybrid	Intensity	Hybrid	Intensity	Hybrid	Intensity	Hybrid
1	1.5	0.2	2.7	0.1	7.7	0.1	3.9	0.2
2	4.5	0.1	2.8	0.2	12.6	0.1	3.8	1.2
3	3.7	0.1	2.7	0.3	6.4	0.1	3.0	0.3
4	2.7	0.2	4.1	0.2	7.1	0.3	3.4	1.6
5	2.2	0.1	4.4	0.3	8.2	0.2	3.9	0.4
6	5.1	0.1	7.2	0.2	8.1	0.1	2.7	0.2
7	2.2	0.1	5.0	0.3	4.7	0.2	5.6	0.2
8	5.5	0.1	5.2	0.4	6.2	0.2	5.4	0.3
9	1.1	0.1	3.3	0.1	7.9	0.2	1.4	0.4
10	3.1	0.2	3.7	0.7	9.7	0.1	3.1	1.4
Average	3.1 ± 1.5	0.1 ± 0.0	4.1 ± 1.4	0.3 ± 0.2	7.9 ± 2.2	0.2 ± 0.1	3.6 ± 1.2	0.6 ± 0.6

## DISCUSSION

We evaluated the performance of hybrid DIR between the planning CT and the CBCT for the pelvic region, compared with intensity-based DIR.

Maria *et al*. evaluated DIR accuracy between the planning CT and CBCT images for the pelvic region using DSC [24]. They reported that the DSCs for the rectum and bladder using intensity-based DIR were ~0.7, which is comparable with the result of this study for intensity-based DIR (e.g. bladder DSC = 0.69). Since it is crowded with soft tissue such as rectum, prostate, seminal vesicles and bladder in the pelvic region, it was difficult to deform despite organ variations (volume changes) using the intensity-based DIR.

Compared with intensity-based DIR, hybrid DIR obtained higher DSCs and smaller COM displacements. Since hybrid DIR could use anatomical information (i.e. the manual contouring on the planning CT and on each CBCT), hybrid DIR might have higher DIR accuracy than intensity-based DIR in the pelvic region, even though there can be large interfractional organ motion. It should be noted that DSC can only evaluate the amount of overlapping volume, and COM displacement can only evaluate the displacement between two structures. That is, it is not well known whether the hybrid DIR can perform the image within the structure accurately. Wognum *et al*. evaluated hybrid DIR accuracy on CT images of *ex vivo* porcine bladders with radiopaque fiducial markers [32]. Their result showed that the contour-based DIR had higher DSC than intensity-based DIR, but the spatial accuracy as assessed from the markers was low for both algorithms. In this study, we only focused on comparison between intensity-based and hybrid DIR. Contour-based DIR, which used only contour information without image intensity, might have a similar DSC value and displacement of COM. However, hybrid DIR has the potential for improving the spatial DIR accuracy, compared with contour-based DIR. In order to clarify the spatial DIR accuracy for hybrid DIR, further studies are needed.

## CONCLUSION

This study evaluated the hybrid DIR algorithm for pelvic CBCT images. Hybrid DIR had a higher DSC value and smaller COM displacement for all structures and all patients, compared with intensity-based DIR.

## FUNDING

This study was supported in part by the Japan Society for the Promotion of Science Grant-in-Aid for Young Scientists (B) (15K19199).

## CONFLICT OF INTEREST

There is no conflict of interest with regard to this manuscript.
